# Bottom-Up Evaluation of the Uncertainty of the Quantification of Microplastics Contamination in Sediment Samples

**DOI:** 10.1021/acs.est.2c01828

**Published:** 2022-07-13

**Authors:** Vanessa Morgado, Carla Palma, Ricardo J. N. Bettencourt da Silva

**Affiliations:** † Centro de Química Estrutural, Institute of Molecular Sciences, Departamento de Química e Bioquímica, Faculdade de Ciências, Universidade de Lisboa, Campo Grande, 1749-016 Lisboa, Portugal; ‡ Instituto Hidrográfico, R. Trinas 49, 1249-093 Lisboa, Portugal

**Keywords:** microplastics, uncertainty, validation, bottom-up approach, FTIR, Poisson-lognormal distribution, Monte Carlo method

## Abstract

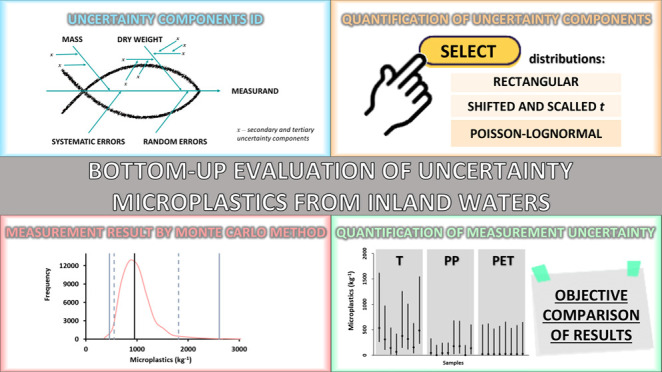

The quantification and comparison of microplastic contamination of sediments are affected by sample heterogeneity and the systematic and random effects affecting sample analysis. The quantification and combination of these components in the measurement uncertainty allows the objective interpretation of analysis results. This work presents the first detailed evaluation of the uncertainty of microplastic contamination quantification in sediments. The random and systematic effects affecting microplastic counts are modeled by the Poisson-lognormal distribution with inputs estimated from duplicate sediment analysis and the analysis of sediments spiked with microparticles. The uncertainty from particle counting was combined with the uncertainty from the determination of the dry mass of the analytical portion by the Monte Carlo method. The developed methodology was implemented in a user-friendly spreadsheet made available as the Supporting Information. The contamination of sediment samples collected in various inland Portuguese waters was determined, ranging from [0; 160] to [361; 2932] kg^–1^ for a 99% confidence level, and compared by assessing if the difference between contamination levels is equivalent to zero for the same confidence level. Several samples proved to have metrologically different microplastic contamination. This work represents a contribution to the objectivity of the assessment of environmental contamination with microplastics.

## Abstract 2

This work describes and applies a novel approach for the bottom-up evaluation of the uncertainty of microplastic contamination quantification in sediments where random and systematic components are quantified and combined.

## 1 Introduction

The presence of large amounts of plastics in the aquatic environment is well recognized and widely debated by the scientific community, governmental bodies, and the population.^1^ Statistics point out that around 75 to 199 million tonnes of plastics are currently contaminating the oceans.^2^ Assuming the mean value of 137 million tonnes and the total area of all oceans (361.9 × 10^6^ km^2^), this contamination level represents an average of 378 kg of plastics in each 32 m × 32 m square of the ocean (approximately two basketball fields). Riverine waters and sediments are a major pathway for plastic litter toward oceans.

Plastic contamination affects the ecosystems differently, depending on their size and shape. Macro-, nano-, and micro-plastics are plastic particles larger than 5 mm, smaller than 1 μm, and with a size between these two limits, respectively.^3^ Microplastic is mistaken for food by smaller organisms being a vehicle for accumulating several contaminants in the food chain.^4^ According to their shape, microplastics can be characterized as fragments, pellets, fibers, or filaments.^4^ The color of particles can also be used to guess their origin since transparent particles are frequently produced from disposable bottles and cups. The micro- and nano-particles can be manufactured with that size or result from the fragmentation of larger particles by environment agents being designated primary or secondary microplastics, respectively.

Research on the levels of microplastic contamination in different environmental compartments worldwide (e.g., rivers, open ocean, estuarine areas, etc.) has been reported.^5^ Typically, the studies on the presence of microplastics on a specific matrix involve the characterization of these particles regarding their chemical (i.e., type of polymer) and physical (i.e., shape, size, and color) properties. However, the uncertainty associated with contamination quantification has not been reported, making the comparison of similar contamination levels impossible.^6,7^ The quality of a measured quantity can be expressed by the measurement uncertainty (MU), which allows assessing if the measurement is fit for purpose and an objective and sound interpretation of the measurement result.

The evaluation of the measurement uncertainty, in particular the quantification of uncertainty components, can follow different approaches that can be divided into “top-down” or “bottom-up”, which provide different knowledge on the measurement quality.

The top-down approaches can be further divided based on interlaboratory data, also known as “supralaboratory” (SLA), and based on in-house validation data, known as the “supra-analytical” (SAA) approach.^8^ In both “top-down” alternatives, the uncertainty sources are grouped in associated with random and systematic effects quantified from precision and trueness tests, respectively. In precision tests, the same sample/s is/are analyzed by different laboratories (SLA) or on different days by the same laboratory (SAA), and in trueness tests, it is assessed the agreement between the reference and the mean measured value from the analysis of a reference or various reference materials.^8−14^ Top-down evaluations are not particularly useful for measurement optimization and involve an overvaluation of the measurement uncertainty by double-counting measurement precision in the evaluation of systematic effects.

On the other hand, in a “bottom-up” or “subanalytical” uncertainty evaluation, whose principles are described in the Guide to the Expression of Uncertainty in Measurement (GUM),^15^ the uncertainty sources should be individually identified and, subsequently, quantified and combined. The quantification of the uncertainty components by this approach can be hampered by model complexity and component correlation. However, bottom-up assessments are particularly useful if measurement uncertainty and cost must be reduced by managing major and minor uncertainty components, respectively. Therefore, the evaluated uncertainty is a function of the measurement performance, the collected performance data, and how uncertainty is modeled from available information.^8^

Counting the number of microplastics of a sample originates discrete quantitative data instead of continuous quantitative data such as the mass of a species. However, after dividing the number of counted particles by the analytical portion, typically a mass or volume, a continuous variable is obtained. Nevertheless, understanding and handling the discrete nature of particle counting is essential for the detailed modeling of the uncertainty of microplastic contamination quantification of environmental samples.

Poisson distribution is adequate to model discrete count variables with a known mean value, λ, where this mean is a rational value larger than zero. However, since the distribution mean is known with some uncertainty, it can be modeled assuming a lognormal distribution with a median equivalent to the best estimate of λ and a standard deviation expressing λ uncertainty.^16^ One of the lognormal distribution features that makes it particularly adequate to model λ is that it produces rational values larger than zero. A Poisson distribution with a mean value modeled or generalized by a lognormal distribution is called Poisson-lognormal.

Poisson and Poisson-lognormal distributions have been used to model microbiological colonies counting in the microbiological analysis of foodstuffs and environmental samples.^17−19^ Poisson regression was used to determine microplastics (e.g., color and shape) and environmental (e.g., total dissolved solids and temperature) characteristics that contributed to microplastic ingestion by African catfish.^20^ The only relevant limitation of this distribution is the mathematical complexity of its application. This limitation can be overcome by the development of user-friendly and validated software.

The generalized Poisson-lognormal distribution complies with all five suitability criteria proposed by I. Jongenburger et al. (2012) for microbiological analysis of foodstuffs. It is a non-negative and discrete distribution that allows zeros values and converges exactly to the Poisson or the Lognormal distribution, assuming appropriate parameters.^16^ Thus, the generalized Poisson-lognormal distribution can be used to model systematic and random components affecting microplastics counting. The uncertainty from the estimated mass of the analytical portion of the sediment, divided into microplastics counting, can be evaluated by combining relevant systematic and random uncertainty components.^12^ The distribution of the ratio between the number of microplastics and the dry mass of sediment can be simulated by the Monte Carlo method (MCM) based on simulated distributions of the inputs. The MCM, described in a supplement to the Guide to the Expression of Uncertainty in Measurement (GUM),^21^ is used to simulate the distribution of an output quantity by determining *N* output values from *N* simulations of the input quantities. The distribution of the output quantity can be characterized by adequate statistical parameters of the simulated values, such as the mode and relevant percentiles.

This work aims at the bottom-up evaluation of the uncertainty of measurements of microplastics in sediment samples where uncertainty components are combined by the MCM. This research also includes the application of the uncertainty models to estimate and compare microplastic contaminations of sediments collected from four Portuguese inland waters. The developed tool was applied to the quantification of (i) total (T) microplastics less dense than an aqueous solution of saturated NaCl, (ii) polypropylene (PP) microparticles with fragment shape, and (iii) poly(ethylene terephthalate) (PET) microparticles independently of the shape, in sediment samples. A user-friendly and validated spreadsheet for quantifying the measurement uncertainty is made available as the Supporting Information. The comparison of microplastic contamination of a pair of sediment samples involved the MCM simulation of the difference in results.

As far as the authors know, this is the first bottom-up evaluation of the uncertainty of microplastic quantification in environmental samples. This work contributes to the objective interpretation of levels and trends of microplastic contamination of environmental samples considered a priority by the scientific community dedicated to this research area.

## 2 Materials and Methods

### 2.1 Sampling and Samples Preparation

**Figure 1:**
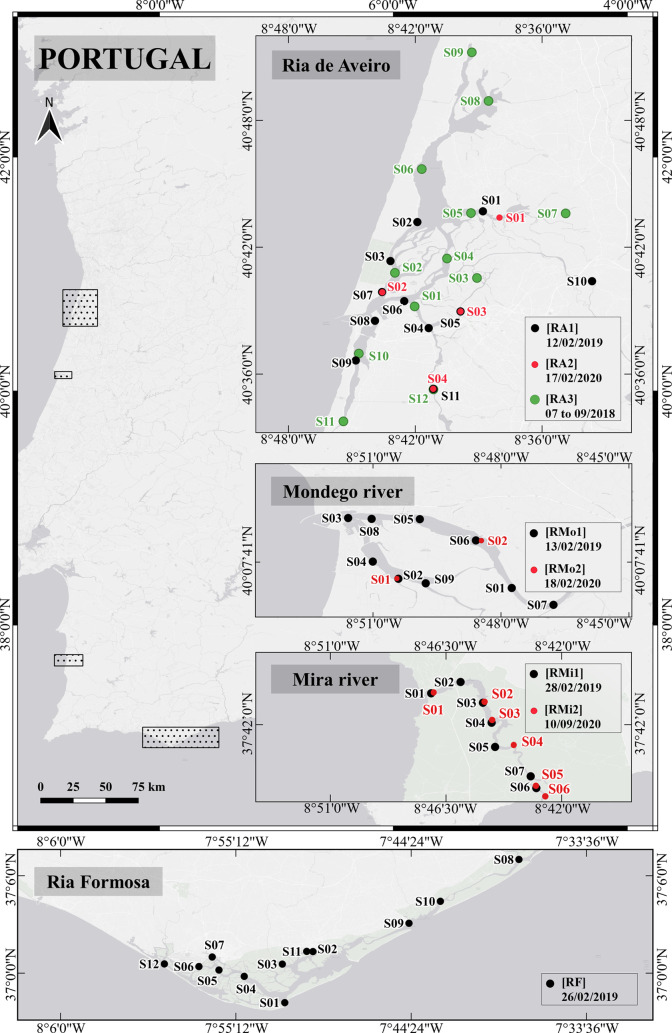
Sampling positions in the aquatic systems map: Ria de Aveiro (RA1; RA2; RA3), Ria Formosa (RF), Mira river (RMi1; RMi2), and Mondego river (RMo1; RMo2). The sampling positions of the various campaigns performed in RA, RMi, and RMo are identified with different colors (black, red, and green).

Sixty-three sediment samples were collected from previously defined locations of Portuguese inland waters, namely, Ria de Aveiro (RA), Ria Formosa (RF), Mondego River (RMo), and Mira River (RMi). RA and RMo are located in the center/north, and RF and RMi at the south of mainland Portugal (Figure 1). Three campaigns in RA (RA1, RA2, and RA3) between July 2018 and February 2020 collected 27 sediment samples. RMo was monitored two times in February 2019 (RMo1) and one year later (RMo2), collecting 11 sediment samples in total. RMi was studied on two occasions in February 2019 (RMi1) and September 2020 (RMi2) through the analysis of 13 sediment samples. RF was monitored in February 2019 through the analysis of 12 sediment samples. The sediment samples from RA2, RMo2, and RMi2 were collected at the same geographical positions as some RA1, RMo1, and RMi1 samples approximately a year later. The approximate coordinates of sample positions can be visualized in Figure 1.

About 0.05 m^2^ and 5 kg sediment area and mass were collected from a boat using a Petite Ponar Grab or a Ponar Grab sampler. The samplers penetrated the bottom by approximately 5 cm, depending on the sediment type.^22^ Samplers were slowly raised to minimize any particle losses from the sample edges.

To minimize the overestimation of microplastic content in sediments by the deposition of additional particles during sample manipulation (analytical contamination), it was cleaned boat deck before collection, reduced the number of people during sample manipulation, homogenized sediment in an aluminum tray using a wooden spoon and covered about 2 kg of sediment in aluminum containers. The transport of samples was carried out using cool boxes to the laboratory. In the laboratory, subsamples of approximately 300 g were transferred to a glass container and frozen below −18 °C until preparation. No blanks test or spikes were performed during sampling.

Sediment samples were prepared after thawing at room temperature for several hours. The summarized analytical procedure is the following: (i) Sediment wet sieving using ultrapure water with a mesh size of 5.6, 5, 2 mm, and 50 μm to isolate sediment matter with the size range of microplastics; (ii) weighing 8.0–100.0 g of sieved sediment in a Mettler PE 1600 analytical balance; (iii) digesting the aliquot using peroxide hydrogen (H_2_O_2_, 30 or 35%, Merck)—the digestion efficiency of 30% or 35% H_2_O_2_ is not significantly different; (iv) separating the lighter microparticles from the denser matter of the samples by mixing with 200 mL of sodium chloride saturated solution (NaCl, 99.5%, AppliChem Panreac) in a measuring cylinder and filtrating the solution above the settled matter to a filter (GF/C, 1.2 μm pore size and Ø = 47 mm, Roth) or polycarbonate membranes (0.45 μm pore size and Ø = 47 mm, Whatman); (v) Repeating the density separation and supernatant filtration of the matter settled in the bottom of the cylinder; (vi) Storing filters in closed Petri dishes before (micro-)ATR-FTIR (Fourier Transform Infrared) identification (see Section 2.2).

A detailed description of the used material, chemicals, and equipment is made available as the Supporting Information.

### 2.2 Microplastics Identification

Two membranes per sediment sample were obtained and handled under stereomicroscope Leica MZ 16 F (115× maximum amplification) set at 10× to 25× amplification to examine and isolate the microparticles suspected to be microplastics. The 150 membranes obtained were entirely examined to minimize the under- or over-estimation of the number of microplastics in the samples, contrary to the suggested by some authors.^23^ The identification of the shape and color of the microplastics were performed under the stereomicroscope following the guidelines proposed by Frias et al..^24^

The isolated suspicious microparticles were mainly analyzed by a PerkinElmer micro-FTIR spectrometer Spotlight 200i Microscope System equipped with a mercury–cadmium–telluride (MCT) detector or by a PerkinElmer Spectrum Two FTIR. The instrumental conditions of the FTIR analysis are described following guidelines proposed by Andrade et al..^25^ The spectra obtained by the micro-FTIR were acquired in reflectance mode from 4000 to 600 cm^–1^ using eight scans or in micro-ATR mode from 4000 to 500 cm^–1^ using four scans to reduce analysis time.^26,27^ For 94% of the particles, a 100 × 100 μm^2^ aperture was used for spectrum acquisition, but the remaining smaller particles were identified using reduced apertures. The four spectra from microparticles with sizes larger than 1000 μm, obtained by the Spectrum Two FTIR, were acquired in universal ATR mode from 4000 to 600 cm^–1^ using eight or four scans.^26,27^ A strong Norton–Beer apodization with 1 and 4 cm^–1^ wavenumber and resolution intervals, respectively, were used in all operational modes. The instrumental signal was acquired in transmittance units (%*T*). The scan of the background was acquired before the analysis.

The infrared spectra were collected by the PerkinElmer Spectrum software version 10.6.2.1159, also used to measure the size of the microplastics by taking the distance between the two farthest points of the microparticle. No further handling (e.g., normalization, background correction, etc.) was considered for spectra identification. Spectra processing and comparison for particle identification were performed using a user-friendly and validated Microsoft Excel spreadsheet developed by Morgado et al.^28^

The validation of the method of microplastics identification, which includes instrumental parameters and spectra comparison algorithms and criteria, was performed as described by Morgado et al.,^28,29^ ensuring true and false identification rates are not lower or higher than 95 or 5%, respectively. After determining the match between micro-FTIR spectra of manually identified microparticles and a reference spectrum of the same polymer, the bootstrap numerical sampling method is used to estimate the match value lower than 95% of the determined match (minimum match value), setting the true positive rate of identifications to 95%. The minimum match is used in future identifications of unknown particles. Afterward, it is assessed if the minimum match is not lower than more than 5% of the match between the reference spectrum and spectra of particles from a different polymer to ensure the false-positive rate is lower than 5%. Different unweighted and weighted match algorithms and spectrum requirements were tested. The false identification of particles in samples used to validate microplastics quantification in sediments impacts on estimated measurement precision and trueness and on the analysis of samples by the validated method. The microplastic identification can be reported with uncertainty as a likelihood ratio determined as the ratio between true- and false-positive rates.^28−30^

### 2.3 Dry Weight Fraction Determination

The dry weight fraction, *W*_d_, of the sediment samples, required to report the number of microplastics present in a dry sample mass, was determined. The *W*_d_ was estimated by drying a sediment portion in an oven at (105 ± 2) °C following EN 12880:2000.^31^Eq 1 presents the expression used to determine *W*_d_.
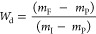
1where *m*_P_ is the mass of the empty Petri dish, *m*_I_ is the initial gross mass of the sample, and *m*_F_ is the dry and final gross mass of the sediment sample.

### 2.4 Performance Assessment and Quality Control

For determining the measurement precision, 12 sediment samples from different inland waters were prepared and analyzed in duplicate: one sample from RA1 and RMi, two samples from RF and RMo2, and six samples from RMi2. To 12 analytical portions of sediment, it was added five films smaller than 3 mm of translucent polyethylene (PE), and five fragments each smaller than 3 mm of green poly(ethylene terephthalate) (PET), translucent polypropylene (PP), and pink polystyrene (PS) before sample preparation to check their recovery during digestion, density separation, and counting, designated “positive controls”. Airborne particles contamination (“analytical contamination”) of areas where samples were manipulated was monitored called “negative controls”.

The quality control measures implemented are described in detail in the Supporting Information. The guidelines proposed by Hermsen et al.^32^ for classifying the extent of quality control measures were followed, after adequate adjustments to sediment analysis, being scored 15 out of 20 points for procedure adequacy. The nonmaximum score results from precision being estimated from duplicate instead of triplicate positive controls, and tests were performed on the few types of studied sediments. The deviation from the defined ideal quality control procedure is not considered relevant or is justified by the defined analytical method scope.

## 3 Evaluation of the Measurement Uncertainty

### 3.1 Definition of the Measurand

The evaluation of the measurement uncertainty starts with the definition of the quantity to be measured, i.e., the measurand, and the algebraic relationship between the measured value and the parameters on which it depends (i.e., the input quantities). The measurand is the number of specific or various types of microplastics in dry mass of the sediment sample. The type of microplastic(s) is defined by the polymer type, particle size, and shape. The way measurand is defined excludes the sampling process from the measurement, being only characterized the sample and not the sampled population.^33−36^ Measurement results are expressed in kg^–1^ units, although some authors prefer to express the same value not following the rules of the International System of Units as “number of particles per kg”.^37^

Three types of particles were quantified in this work: (i) microplastics less dense than saturated NaCl solution—T; (ii) microfragments of polypropylene—PP, and (iii) microparticles of polyethylene terephthalate with different shapes—PET.

### 3.2 Identification of the Uncertainty Components

The second stage of measurement uncertainty evaluation is the identification of the sources of uncertainty that contribute with random and systematic effects to the measured quantity. These effects are responsible for possible deviations between the measured and conventional “true value” of the measurand. The random errors vary with replicate measurements typically following known distribution models. On the other hand, the systematic effects produce error components that remain constant or vary in a predictable manner in the measurement interval, with the time, type of analyzed item, or another factor.

**Figure 2:**
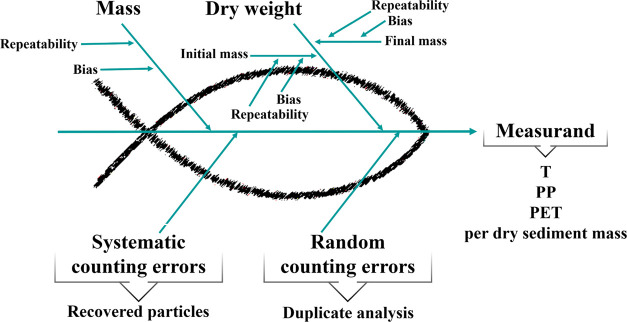
Identification of the uncertainty sources throughout an Ishikawa diagram, also known as fishbone diagram.

Figure 2 presents an Ishikawa diagram, also known as a cause/effect or “fishbone” diagram, that graphically represents the identified uncertainty sources as arrows or “spine” links to the same backbone. This representation helps avoid forgetting or double counting uncertainty sources or any correlation.

### 3.3 Quantification of the Uncertainty Components

#### 3.3.1 Random Errors

The random errors, ε, were modeled from the measured values of 12 duplicate samples collected from the distinct inland water compartments: RMo1, RA1, RMi1, RF, and RMi2. The difference in the number of T, PP, and PET between duplicate samples was determined depending on the studied measurand. Since the number of microplastics in a sample is a discrete variable, the probability mass function (PMF) of Poisson distribution, presented by eq 2, was recognized to be an adequate model to describe the random effects affecting microplastics analysis.
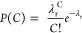
2where *C* is the number of particles (natural number, {0, 1, 2, 3, (...)}), *P*(*C*) is the mass probability of observing *C* number of particles in a sample, *e* is the Euler’s number, and λ_r_ is the mean of observed particle counting (fractional number larger than zero; the index “r” stands for “random”).

If the studied sediment is analyzed several times, λ_r_ would be the mean of particles counted in equivalent portions of the sediment. One of the particularities of this distribution is that it does not presume any variability of λ_r_, and assumes that λ_r_ is equal to the expected value, E(*C*), and variance, Var(*C*), of the counts. Since the estimated λ_r_ has its own uncertainty, the previous model was fed with an uncertain λ_r_ value with expected E(λ_r_) value and variance Var(λ_r_) producing a generalized Poisson distribution. In this work, the considered generalization assumes λ_r_ has a lognormal distribution with impossible zero value and a wide distribution tail above E(λ_r_). The resulting distribution is called “Poisson-lognormal”.^38^

For modeling the random effects affecting the determination of particles counts, it was assumed E(λ_r_) is equal to the number of particles observed in the analyzed sample (i.e., E(λ_r_) = *C*), and Var(λ_r_) is estimated from duplicate counting observed in duplicate analyses of sediment samples. The variance, Var(*d*(*C*)), of the difference of *C*, *d*(*C*), observed in samples is used to estimate Var(λ_r_)(Var(λ_r_) = Var(*d*(*C*))/2) since it is known that the variance of the difference of two variables with Poisson distribution is 2 times larger than the variance of individual counts.^39^ The random effects considered in this calculation comprise all analytical steps, including those associated with the measurement of the mass of the analytical portion, although this contribution should be negligible.

To simulate the Lognormal distribution of λ_r_, it is necessary to calculate the expected value E(ln(λ_r_)) (i.e., the mean value), and the variance Var(ln(λ_r_)) of the natural logarithm of λ_r_, i.e., ln (λ_r_). Eqs 3 and 4 are the well-known formulas to calculate E(ln(λ_r_)) and Var(ln(λ_r_)),^40^ respectively.
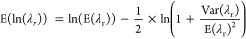
3
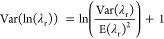
4

The simulated values of the normal distribution with mean E(ln(λ_r_)) and variance Var(ln(λ_r_)), Normal (E(ln(λ_r_)); Var(ln(λ_r_))), are converted into simulated λ_r_ with lognormal distribution, Lognormal (E(λ_r_); Var(λ_r_)), by determining the natural exponent of each simulated ln(λ_r_). The simulated *C* values are microplastic counts not corrected for losses in sample preparation but expressing random effects. The λ_r_ values were simulated 49 999 times, its distribution being characterized by the 0.5th and 99.5th percentiles (*P*0.5 and *P*99.5, respectively).

#### 3.3.2 Systematic Errors

The systematic effects affecting microplastics counting were quantified by analyzing 12 samples fortified with a known quantity of microplastics. Five particles of PE, PET, PP, and PS (see Section 2.4) were added to sediment samples before preparation and, subsequently, subject to the procedure described in Sections 2.1 and 2.2. Photos of the added particles were taken to facilitate their identification by the characteristic shape and color in recovery tests.

The systematic component of counts error, , was estimated by multiplying the original sample count, *C*, by factor *F* ( = *C*·*F*) representing the opposite of the observed mean counts error (*N* – *n̅*) normalized by the mean number of recovered particles, *n̅* (eq 5).
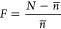
5where *N* is the number of added particles (i.e., 12 × 5 = 60 or 12 × 15 = 180 in this study, depending on studies particle(s) type(s)). The corrected counts, *C*_C_, are estimated by adding  to the original count (i.e., *C*_C_ = *C* + *C*·*F*). The variance of recovered particle counts, Var(*n*), was calculated to express their dispersion.

Analogous to the study of random effects, the systematics effects were modeled by the Poisson-lognormal distribution that considers the uncertainty of systematic effects determination. In this case, the expected value of the systematic error,  or E(λ_s_), varies with the *C* (i.e., E(λ_s_) = *C*·*F*) (where the “s” in λ_s_ stands for “systematic”). The variance Var(λ_s_) is estimated by eq 6 that applied the Law of the Propagation of Uncertainty (LPU) to  = *C*·*F*, knowing that *N* is a constant without uncertainty since the added number of particles is known without any doubt. In this equation, *C* is also a constant used to convert *F* into .
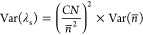
6where Var(*n̅*) is the variance of mean *n*, *n̅* (Var(*n̅*) = (Var(*n*))/*M*) being *M* the total number of *n* estimates. The  is assumed to be lognormally distributed, and the expected value E(ln(λ_s_)) and variance Var(ln(λ_s_)) of the natural logarithm of λ_s_ (i.e., ln(λ_s_)) calculated using eqs 3 and 4, where λ_r_ is changed for λ_s_. The ln(λ_s_) was simulated 49999 times, being its distribution characterized by *P*0.5 and *P*99.5 percentiles.

#### 3.3.3 Weighing

The uncertainty associated with the conventional mass, *m*,^41^ determined in weighing by difference of sediment analytical portion was simulated by the MCM. Eq 7 represents the equivalent version of components combination by the LPU.
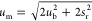
7where *u*_m_ is the standard uncertainty of *m*, and *u*_b_ and *s*_r_ are the standard uncertainty associated with systematic effects and the standard deviation of mass measurements repeatability, respectively. These terms are counted twice, assuming the same value for both the tare and gross masses. The *u*_b_ value is quantified by the maximum admissible error, *E*_M_, considered in balance calibration assuming a rectangular distribution.^12^ To simulate the measurement repeatability components, it was considered a scaled and shifted *t* distribution for the degrees of freedom of *s*_r_.

The uncertainty of the differences (*m*_F_ – *m*_P_) and (*m*_I_ – *m*_P_) considered in the determination of *W*_d_ of the sediment sample is simulated equivalently to *m*. In the evaluation of *W*_d_ uncertainty, it is assumed the drying process in the oven is responsible for a negligible uncertainty component.

### 3.4 Combination of Uncertainty Components

The uncertainty components were combined by the MCM applicable to both continuous and discrete variables. Eq 8 describes how simulated values of *m*, *W*_d_, ε, and  were combined to estimate corrected microplastic counts per dry sediment mass, ω.
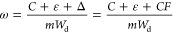
8where (*C* + ε) are simulated combined as described in Section 3.3.1, and Δ and *mW*_d_ are simulated as described in Sections 3.3.2 and 3.3.3, respectively.

A user-friendly MS Excel spreadsheet, made available as the Supporting Information, was developed to combine the uncertainty components and estimate microplastic contamination in sediments with uncertainty. This tool simulates and combines random and systematic errors in microplastic counts and the dry mass of the analytical portion. The distribution of ω was estimated from 49 999 simulations. The first sheet of the spreadsheet presents use instructions.

The mode of simulated ω represents the most probable or the best estimate of the value of the measurand. *P*0.5 and *P*99.5 were used to define the confidence interval for 99% confidence level, which has a 99% probability of including the true value of the measurand.

**Figure 3:**
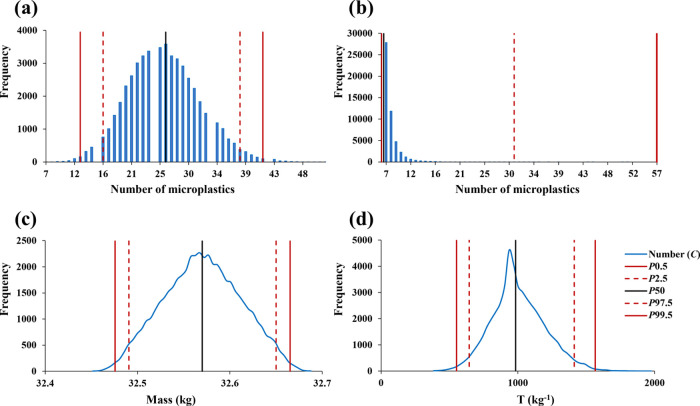
Simulated distribution and percentiles of uncertainty components and final result of the determination of T in sample S01 of the first sampling campaign of Ria de Aveiro (RA1): (a) random error in particles count centered in the observed count, *C* + ε; (b) systematic error in particles count, ; (c) dry mass of the analytical portion, *mW*_d_; and (d) T per dry mass of sediment, ω.

Figure 3 presents the simulated distribution and percentiles of uncertainty components and final results of the determination of T microplastics in sample S01 collected in the first campaign of Ria de Aveiro (RA1): (a) random error in particles count centered on the observed count, *C* + ε; (b) systematic error in particles count, ; (c) dry mass of the analytical portion, *mW*_d_; and (d) T microplastics per dry sediment mass, ω. The distribution of *mW*_d_ (Figure 3c) has a triangular shape, although the systematic effects components were modeled using a rectangular distribution. The simulated distribution of T in sample S01[RA1] (Figure 3d) is continuous and rather complex.

## 4 Results and Discussion

The 12 sediments analyzed in duplicate (24 tests) presented quantitative (i.e., nonzero) microplastic contaminations. For the determination of the studied three parameters (i.e., T, PP, and PET), the variance of counts differences, Var(*d*(*C*)), equals to 12.0, 2.50, and 0.967, estimating a random error variance, Var(λ_r_), of 5.98, 1.25, and 0.483, respectively (i.e., Var(*d*(*C*))/2). From the analysis of 12 fortified samples with five PE, PET, PP, and PS particles, it was observed mean numbers of recovered particles, *n̅*, of 3.97, 3.58, and 1.67 with a variance, Var(*n*), of 0.0372, 0.143, and 0.505, for T (PE + PP + PS), PP, and PET, respectively. The recovery of T was estimated as the combined recovery of PE, PP, and PS since the studied polymers are less dense than saturated NaCl solution.

**Figure 4:**
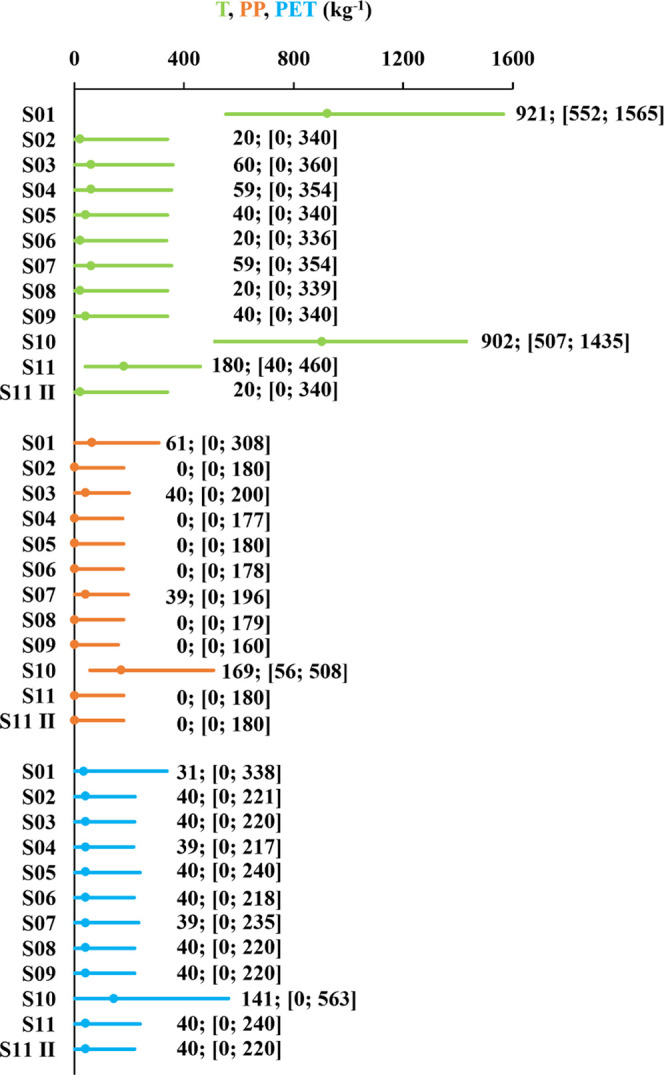
Mode and confidence interval, for 99% confidence level, of simulated estimates of microplastic contamination (T—microplastics less dense than saturated NaCl solution, PP—microfragments of polypropylene, and PET—poly(ethylene terephthalate) with different shapes) of the samples collected in the first campaign of Ria de Aveiro (RA1). Confidence limits are defined by the 0.5th and 99.5th percentiles (*P*0.5 and *P*99.5, respectively). The mean values of T, PP, and PET determinations are reported, with uncertainty, in the first line of values of Table 1.

Together with the weighing performance parameters, the described performance parameters were used to evaluate the uncertainty of microplastics quantification (T, PP, and PET) in the sixty-three sediments samples collected from the studied environmental compartments (i.e., RA, RF, RMi, and RMo). Figure 4 presents the simulated mode and uncertainty interval, for 99% confidence level, of results from the analysis of T, PP, and PET in samples collected in RA1. And 99% instead of 95% confidence levels were considered since in the latter, on average, one in 20 confidence intervals will not include the true value of the measured quantity. The simulated mode is the best estimate of the contamination level, and the uncertainty is expressed by *P*0.5 and *P*99.5. It was observed that the modes of T and PET in RA1 samples vary between 20 and 921 kg^–1^ and between 31 and 141 kg^–1^, respectively. Regarding the presence of PP, the mode varied between 0 and 169 kg^–1^. The simulated estimate of the measurand has a rather asymmetric distribution with long right tails making the mode different from the mean value. In most cases, the *P*0.5 is zero and occasionally coincides with the mode. A *P*0.5 equal to zero indicates the estimated microplastic contamination is close to the level where it is difficult to distinguish from no contamination. When the mode is zero, the level is not distinguishable from no contamination. Figures S1–S7 in the Supporting Information present the mode and uncertainty of microplastics quantification for the remaining campaigns and studied three parameters.

**Figure 5:**
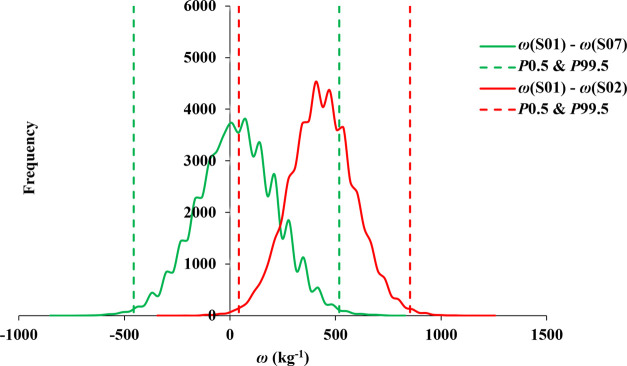
Simulated differences of estimated microplastic contamination of samples S01 and S07, and S01 and S02 from the first sampling campaign of Mira river (RMi1). The *P*0.5 and *P*99.5 percentiles of the simulated differences are reported, concluding that S01 and S07 are equally contaminated since *P*0.5 ≤ 0 ≤ *P*99.5 and S01 is more contaminated than S02 since *P*0.5 > 0. To extend the contamination difference conclusion to a larger area around sampling points, the sampling uncertainty must be considered.

To compare the microplastic contamination level of a pair of samples, it was simulated the difference in estimated microplastic contamination by the MCM since it takes the complex distribution of estimated values into account. Figure 5 presents the simulated difference of microplastic contamination observed in RMi1 samples S01 and S07, and samples S01 and S02. In the first and second cases, the zero value is within and beyond the interval limited by *P*0.5 and *P*99.5, indicating that the sample contaminations are metrologically equivalent or different for 99% confidence level, respectively. Sample S01 is more contaminated than sample S02 since *P*0.5 is larger than zero. When *P*99.5 is smaller than zero, the second sample of the difference is less contaminated. The simulated differences from Figure 5 are rather symmetry although noisy due to the low number of simulations considered. Nevertheless, the simulated percentiles do not vary significantly with the simulation run.

However, since the estimated microplastic contamination of sediments does not consider the sampling uncertainty, this comparison is not representative of a larger fraction of river sediments.

Although the evaluation of the uncertainty of microplastic contamination estimated for each sample is important, since it objectively informs on the expected true value of the contamination, it is in the comparison of the contamination of two samples that this concept is particularly relevant. If a maximum threshold for microplastic contamination is defined, the uncertainty from the sample analysis could be used to assess the conformity with that limit.^42−44^

The microplastic contaminations of sediment samples collected in each compartment and sampling campaign were compared. The contaminations of samples collected in the same GPS coordinates in different campaigns were also compared. The results from these comparisons are made available as the Supporting Information. In this work, it was also simulated the mean of the estimated microplastic contamination of all samples collected from the same environmental compartment and sampling occasion to allow mean comparisons by calculating the difference of the means. The meaning of the mean contamination is even more difficult to describe since it can be not representative of the heterogeneity of compartment contamination.

Table 1 presents the simulated mean values of microplastic contamination observed in the various sampling campaigns, associated with uncertainty for a 99% confidence level, for the studied parameters: T, PP, and PET. Table 2 presents the outcome of the comparison of mean values from the simulation of their differences. The first (1st), second (2nd), and third (3rd) subcolumns are associated with T, PP, and PET values, respectively. This table shows that most of the mean values of the various sampling campaigns are equivalent (Eq) independently of the type of contamination (T, PP, or PET). Nevertheless, the mean T of the samples of RMi1 is larger than for RMo1, RMi2, RA1, RA2, and RF. Besides that, the mean T of RMo1 and RMi2 is lower than the mean of RA3 and RMo2. Also, the mean T of RMo1 is lower than that for RA1, and the mean T’s of RA2 and RF are lower than of RMo2.

**Table 1:** Mode and Confidence Interval, for 99% Confidence Level, of Simulated Mean Values of Estimated Microplastic Contamination of Sediments Determined for the Various Sampling Campaigns^a^^b^^c^^d^

	T (kg^–1^)	PP (kg^–1^)	PET (kg^–1^)
[RA1]	227; [165; 309]	53; [26; 93]	69; [37; 118]
[RF]	193; [130; 286]	125; [79; 196]	76; [41; 132]
[RMi1]	357; [272; 466]	107; [64; 169]	66; [27; 120]
[RMo1]	118; [82; 172]	70; [45; 104]	39; [19; 69]
[RMi2]	100; [57; 220]	40; [13; 101]	87; [45; 160]
[RA2]	134; [60; 279]	15; [0; 99]	54; [15; 148]
[RA3]	300; [172; 522]	68; [21; 181]	150; [77; 288]
[RMo2]	380; [220; 599]	130; [50; 260]	70; [10; 240]

a The results are expressed as follows: “mode; [P0.5; P99.5].

b T—microplastics less dense than saturated NaCl solution.

c PP—microfragments of polypropylene.

d PET—poly(ethylene terephthalate) with different shapes.

**Table 2:**
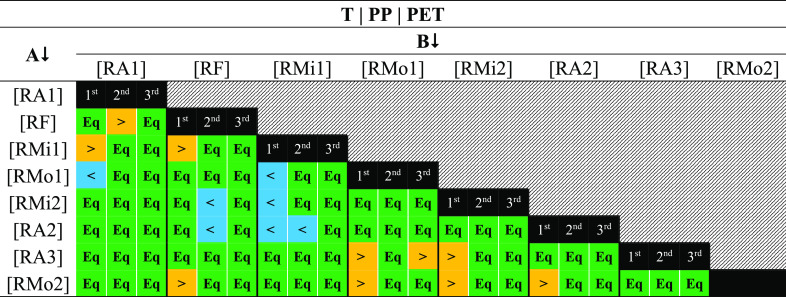
Outcome of the Comparison between Mean Contaminations Estimated in the Various Sampling Campaigns^a^^b^^–^g

a First, second, and third subcolumns are associated with the determination of T, PP, and PET, respectively.

b Eq—Equivalent.

c >—A greater than B.

d <—A lower than B.

e T—microplastics less dense than saturated NaCl solution.

f PP—microfragments of polypropylene.

g PET—polyethylene terephthalate with different shapes.

Regarding the contamination level of PP, the mean value of RF samples is greater than the mean of RMi2, RA1, and RA2. Nevertheless, the mean value of RA2 is lower than for the RMi1. Microplastic contamination on RA result from industrial and agricultural sources, RMo tide influence, RMi agricultural activities, and RF urban sites proximity.

The tables with the comparison of the microplastic contamination of samples collected from the same sampling campaign or collected from the same coordinates in different campaigns are made available as the Supporting Information (Tables S1–S33). Only the samples of RA2 campaign present equivalent T values. Nevertheless, in the other campaigns, some samples have significantly different contamination levels. For instance, S01 and S10 of RA1 are equivalently contaminated but more contaminated than the remaining samples of the same campaign (see Table S1). Regarding the samples collected from the same coordinates, most samples have equivalent T levels, except for S04 of RMi1, which is more contaminated than S03/S03II of RMi2 (see Table S9). In this case, sediment contamination was reduced over time. To infer the representativeness of samples and of this conclusion about sediments contamination, the microplastic contamination heterogeneity and sampling uncertainty should be considered.

Concerning PP contamination, most of the samples from the same sampling campaign present equivalent contamination levels, except for some samples of RMo1 and RF. The S07 of RF has a contamination level equivalent to the samples S02 and S05, and a contamination level lower than the sample S11 of the same campaign. Nevertheless, S11 is only equivalent to sample S02. Besides, S07 and S11 of RF have a contamination level greater than the remaining samples (see Table S15). The S09 of RMo1 has a contamination level greater than S01/S01II, S02, and S06, and S07 is more contaminated than S01 of the same campaign (see Table S18). The samples collected at the same coordinates on different occasions have equivalent contamination levels of PP.

Regarding the PET contamination, it was observed that all samples from the same campaign, mean values of various campaigns, and the samples collected from the same coordinates on different occasions have equivalent contamination levels.

The nonequivalence conclusion of the contamination levels of T, PP, or PET of various samples cannot be extrapolated to a large and representative fraction of the studied environmental compartments since it must be considered the spatial contamination heterogeneity and the sampling uncertainty for this assessment.^33−36^ Nevertheless, since these uncertainty components add uncertainty to the comparisons, the contamination equivalence can describe the reality for the environmental compartment. The justification of observed contamination differences from the characterization of contamination sources and of the aquatic system is beyond the scope of this work.

Since the developed measurement uncertainty models were based on duplicate analysis and spiked samples analysis of some samples and particles, it assumes collected performance data is representative of the other studied samples and particles. More performance data need to be collected to assess the adequacy of this assumption.

The equivalence of microplastic contamination in another study supported by measurement uncertainty evaluation described in this work is also binding.

The quantification of microplastic contamination of sediments with uncertainty allowed, for the first time known to the authors, the objective comparison of contamination levels of various sediment samples. When different samples from different environmental compartments are equally contaminated, the same can be concluded about a larger sediment area around the sampling location. However, indicated contaminations differences considering the uncertainty of the difference must be further investigated for sample representativeness. In this work, the contamination comparisons were performed for a 99% confidence level, associated with a 1% probability of falsely concluding that equivalent sample contaminations are different.

The high uncertainty values observed in some sample results and their comparisons suggest that when this information is not considered, analysts can conclude wrongly about the relevance of observed contamination differences and trends. Therefore, this work is particularly relevant for providing the tool to allow the correct interpretation of the very relevant and large amount of data on microplastic contamination that was and is being collected for the management of the risk of this contamination type.

The developed measurement uncertainty evaluation method and MS Excel tool can be used directly in the determination of microplastic contamination of samples whose analytical portion is dried weight (e.g., soil). After some adaptation, this tool can also be used to characterize analytical portions determined by volume, such as water of different types and origins. Results with uncertainty from microplastic contamination of water and sediments from the same location can be used to better understand how different microplastics distribute in the environment.

## Supporting Information Available

Materials, chemicals, and equipment; quality control and quality assurance of tests; and estimated microplastic contamination of analyzed sediment with uncertainty for 99% confidence level (PDF)
Comparison of estimated microplastic contaminations for 99% confidence level (XLSX)
User-friendly MS-Excel file used for measurement uncertainty evaluation (ZIP)
