# Supplementary material for: Bottom-Up Evaluation of the Uncertainty of the Quantification of Microplastics Contamination in Sediment Samples
Source: Environ Sci Technol. 2022 Jul 13;56(15):11080–90. doi: 10.1021/acs.est.2c01828 (PMC9778736; doi:10.1021/acs.est.2c01828)
Supplement: Supplementary file 1 — es2c01828_si_001.pdf [file es2c01828_si_001.pdf]

Supporting Information

Bottom-up evaluation of the uncertainty of the quantification of microplastics contamination in sediment samples

Vanessa Morgado[ab], Carla Palma[b], Ricardo J. N. Bettencourt da Silva[a]*
[a] Centro de Química Estrutural, Institute of Molecular Sciences, Departamento de Química e Bioquímica, Faculdade de Ciências, Universidade de Lisboa, Campo Grande, 1749-016 Lisboa, Portugal
[b] Instituto Hidrográfico, R. Trinas 49, 1249-093 Lisboa, Portugal
* Corresponding author. E-mail: rjsilva@fc.ul.pt

Content

A. Materials, chemicals, and equipment

B. Tests quality control and quality assurance

C. Figures:

Figure S1. Mode and confidence interval, for 99% confidence level, of simulated estimates of microplastic contamination (T – microplastics less dense than saturated NaCl solution, PP – micro-fragments of polypropylene and PET – polyethylene terephthalate with different shapes) of the samples collected in the second campaign of Ria de Aveiro (RA2). Confidence limits are defined by the 0.5th and 99.5th percentiles (*P*0.5 and *P*99.5, respectively).

Figure S2. Mode and confidence interval, for 99% confidence level, of simulated estimates of microplastic contamination (T – microplastics less dense than saturated NaCl solution, PP – micro-fragments of polypropylene and PET – polyethylene terephthalate with different shapes) of the samples collected in the third campaign of Ria de Aveiro (RA3). Confidence limits are defined by the 0.5th and 99.5th percentiles (*P*0.5 and *P*99.5, respectively).

Figure S3. Mode and confidence interval, for 99% confidence level, of simulated estimates of microplastic contamination (T – microplastics less dense than saturated NaCl solution, PP – micro-fragments of polypropylene and PET – polyethylene terephthalate with different shapes) of the samples collected in Ria Formosa (RF). Confidence limits are defined by the 0.5th and 99.5th percentiles (*P*0.5 and *P*99.5, respectively).

Figure S4. Mode and confidence interval, for 99% confidence level, of simulated estimates of microplastic contamination (T – microplastics less dense than saturated NaCl solution, PP – micro-fragments of polypropylene and PET – polyethylene terephthalate with different shapes) of the samples collected in the first campaign of Mondego River (RMo1). Confidence limits are defined by the 0.5th and 99.5th percentiles (*P*0.5 and *P*99.5, respectively).

Figure S5. Mode and confidence interval, for 99% confidence level, of simulated estimates of microplastic contamination (T – microplastics less dense than saturated NaCl solution, PP – micro-fragments of polypropylene and PET – polyethylene terephthalate with different shapes) of the samples collected in the second campaign of Mondego River (RMo2). Confidence limits are defined by the 0.5th and 99.5th percentiles (*P*0.5 and *P*99.5, respectively).

Figure S6. Mode and confidence interval, for 99% confidence level, of simulated estimates of microplastic contamination (T – microplastics less dense than saturated NaCl solution, PP – micro-fragments of polypropylene and PET – polyethylene terephthalate with different shapes) of the samples collected in the first campaign of Mira River (RMi1). Confidence limits are defined by the 0.5^{th} and 99.5^{th} percentiles (*P*0.5 and *P*99.5, respectively).

Figure S7. Mode and confidence interval, for 99% confidence level, of simulated estimates of microplastic contamination (T – microplastics less dense than saturated NaCl solution, PP – micro-fragments of polypropylene and PET – polyethylene terephthalate with different shapes) of the samples collected in the second campaign of Mira River (RMi2). Confidence limits are defined by the 0.5^{th} and 99.5^{th} percentiles (*P*0.5 and *P*99.5, respectively).

A. Materials, chemicals, and equipment

Sediment samples were sieved using sieves with a metal mesh size of 5 mm, 5.6 mm or 2 mm, and 50 μm. The water content of samples was removed by the Labconco Lyph Lock 1L or Unicryo MC-4L-60 °C freeze-dryer, or using a Memmert UE300 drying oven. Mettler PE 1600 and Mettler AG245 analytical balances were used for the weighing of analytical portions of sediment to be digested and for the determination of the dried weight, respectively. MilliQ water was obtained by the Q POD® Element (Merck Millipore). Ultra-pure water (MilliQ, 18.2 MΩ.cm) was used for the preparation of the saturated sodium chloride (NaCl, 99.5%, AppliChem Panreac) and hydrogen peroxide (H_2O_2, 30% or 35%, Merck) solutions and to rinse and wash steps. Glass beakers and columns were used during sample digestion and density separation, respectively. Glass filtration kit was used for under vacuum filtration of the NaCl solution after density separation. NaCl solution was filtrated to glass fibre membranes (GF/C, 1.2 μm pore size and Ø = 47 mm, Roth) or polycarbonate membranes (0.45 μm pore size and Ø = 47 mm, Whatman). The membranes were stored inside labelled and closed Petri dishes until analysis. Metal tweezers were used to transfer the microparticles suspicious to be microplastics from the glass fibre or polycarbonate membranes to a nitrocellulose membrane or a gold-plaque, which worked as a substrate for the micro-FTIR analysis. The membranes were handled under stereomicroscope Leica MZ 16 F (115× maximum amplification) set at 10× to 25× amplification, where the visual analysis of the colour and shape of microparticles occurred. The isolated suspicious microparticles were mainly analysed by micro-FTIR spectrometer Spotlight 200i Microscope System (Perkin Elmer) equipped with a Mercury–Cadmium–Telluride (MCT) detector or by the coupled Spectrum Two FTIR. The Mercury–Cadmium–Telluride (MCT) detector was cooled with Liquid nitrogen (Air Liquide Portugal). Infrared spectra were collected by the Spectrum software version 10.6.2.1159 (PerkinElmer).

B. Test quality control and quality assurance

The reliability of the results is ensured through the implementation of quality control and quality assurance measures, which allowed better control of the measurements and the reduction of contaminations that can under- or overestimate microplastic contamination. These measures were applied to the equipment, materials and measurement procedure. The two analysts involved in the analysis wore a white cotton lab coat and handled the samples with blue nitrile gloves.
Milli-Q water was used to wash and rinse all material before use. The material was kept in clean air conditions or properly covered before use. Aluminium foil was used to cover the material.

Current laboratory glassware was used whenever possible: volumetric flask for solutions preparation, beakers for the digestion, measuring cylinders for density separation, filtration kit, sticks, and Petri dishes. Metallic materials were also used, such as sieves, tweezers, and spatula.
The airborne contamination was controlled by handling the samples under a fume hood and covering them with aluminium foil or glass whenever they were not being handled (*e.g.*, during digestion). At the end of the digestion, the beakers were washed to the 50 µm sieve to avoid losses of microplastics. NaCl solution was filtrated before use, avoiding any airborne contamination while it was prepared.
Additionally, negative and positive controls were implemented during sample processing. Six negative controls were used to monitor the airborne contamination through blank filters. The filters were placed close to the samples and exposed to the air as many times as samples. The analysis of the blank filters was the same as the samples. The particles found in the negative controls suggested that airborne contaminations were negligible since they were few and distinguishable from the particles observed in the samples. The positive controls were used to provide data on the procedure efficiency by spiking 12 samples with well-known microplastics. This information was used in the quantification of systematic effects for the evaluation of the measurement uncertainty. A well-known number of particles of polyethylene (PE), polyethylene terephthalate (PET) and polypropylene (PP), and polystyrene (PS) were added to the sediment samples before digestion. These spiked samples were processed similarly to the remaining samples. The added particles were easily identified and distinguished from the original microparticles of the samples due to their distinct shape and colour.

C. Figures:

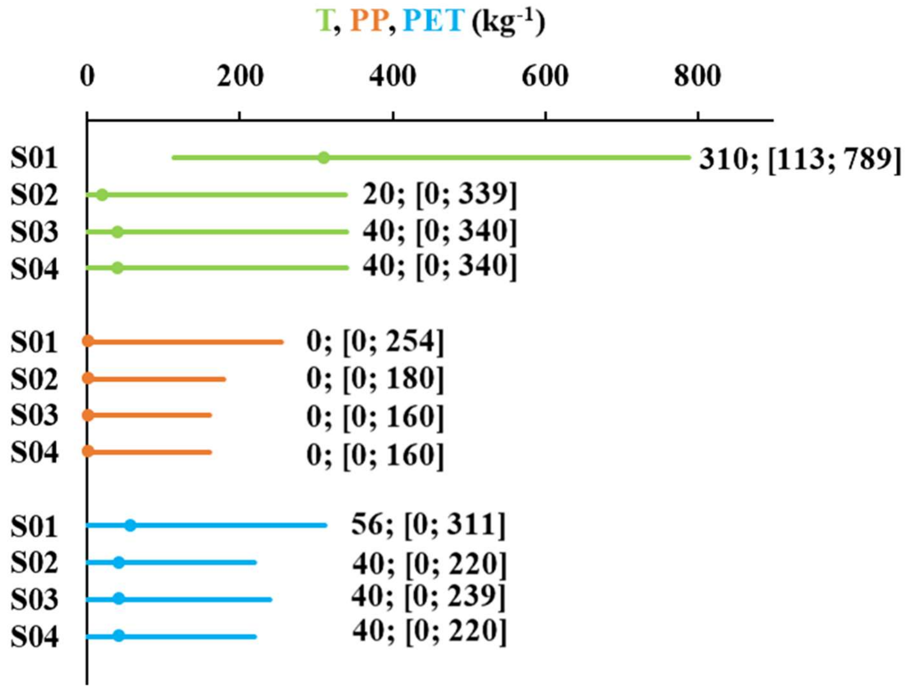

Figure S1. Mode and confidence interval, for 99% confidence level, of simulated estimates of microplastic contamination (T – microplastics less dense than saturated NaCl solution, PP – micro-fragments of polypropylene and PET – polyethylene terephthalate with different shapes) of the samples collected in the second campaign of Ria de Aveiro (RA2). Confidence limits are defined by the 0.5th and 99.5th percentiles (*P*0.5 and *P*99.5, respectively).

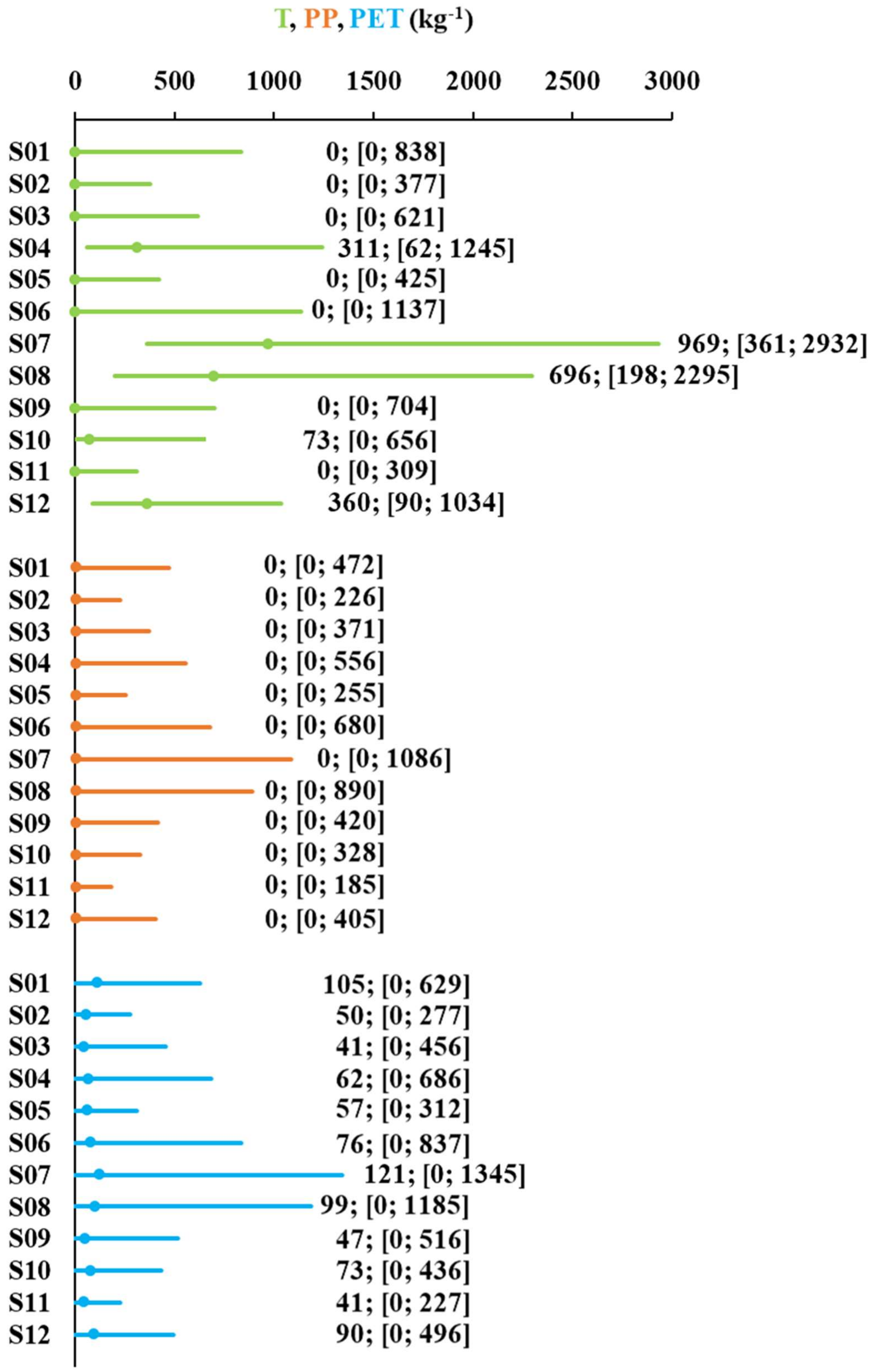

Figure S2. Mode and confidence interval, for 99% confidence level, of simulated estimates of microplastic contamination (T – microplastics less dense than saturated NaCl solution, PP – micro-fragments of polypropylene and PET – polyethylene terephthalate with different shapes) of the samples collected in the third campaign of Ria de Aveiro (RA3). Confidence limits are defined by the 0.5^{th} and 99.5^{th} percentiles (*P*0.5 and *P*99.5, respectively).

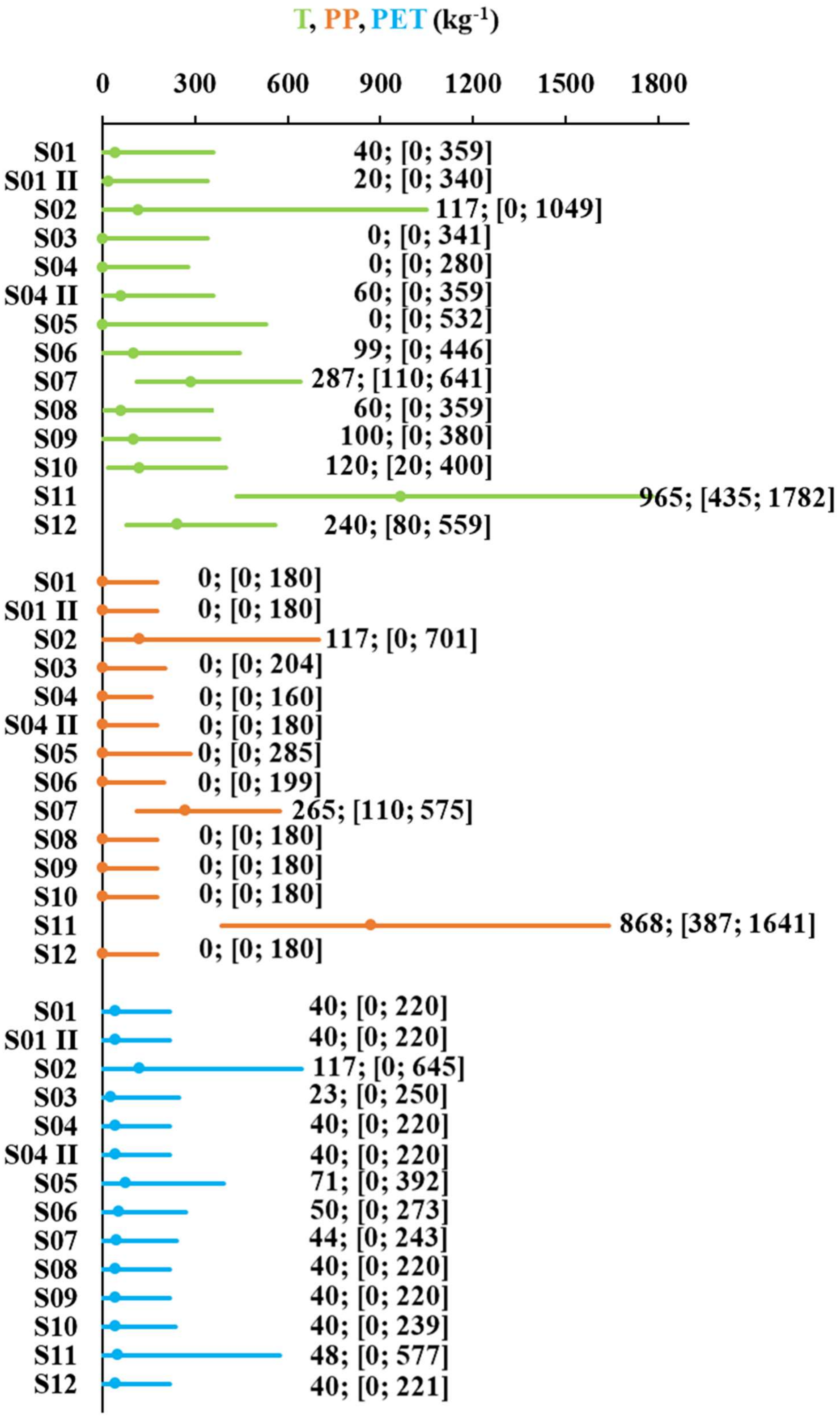

Figure S3. Mode and confidence interval, for 99% confidence level, of simulated estimates of microplastic contamination (T – microplastics less dense than saturated NaCl solution, PP – micro-fragments of polypropylene and PET – polyethylene terephthalate with different shapes) of the samples collected in Ria Formosa (RF). Confidence limits are defined by the 0.5th and 99.5th percentiles (*P*0.5 and *P*99.5, respectively).

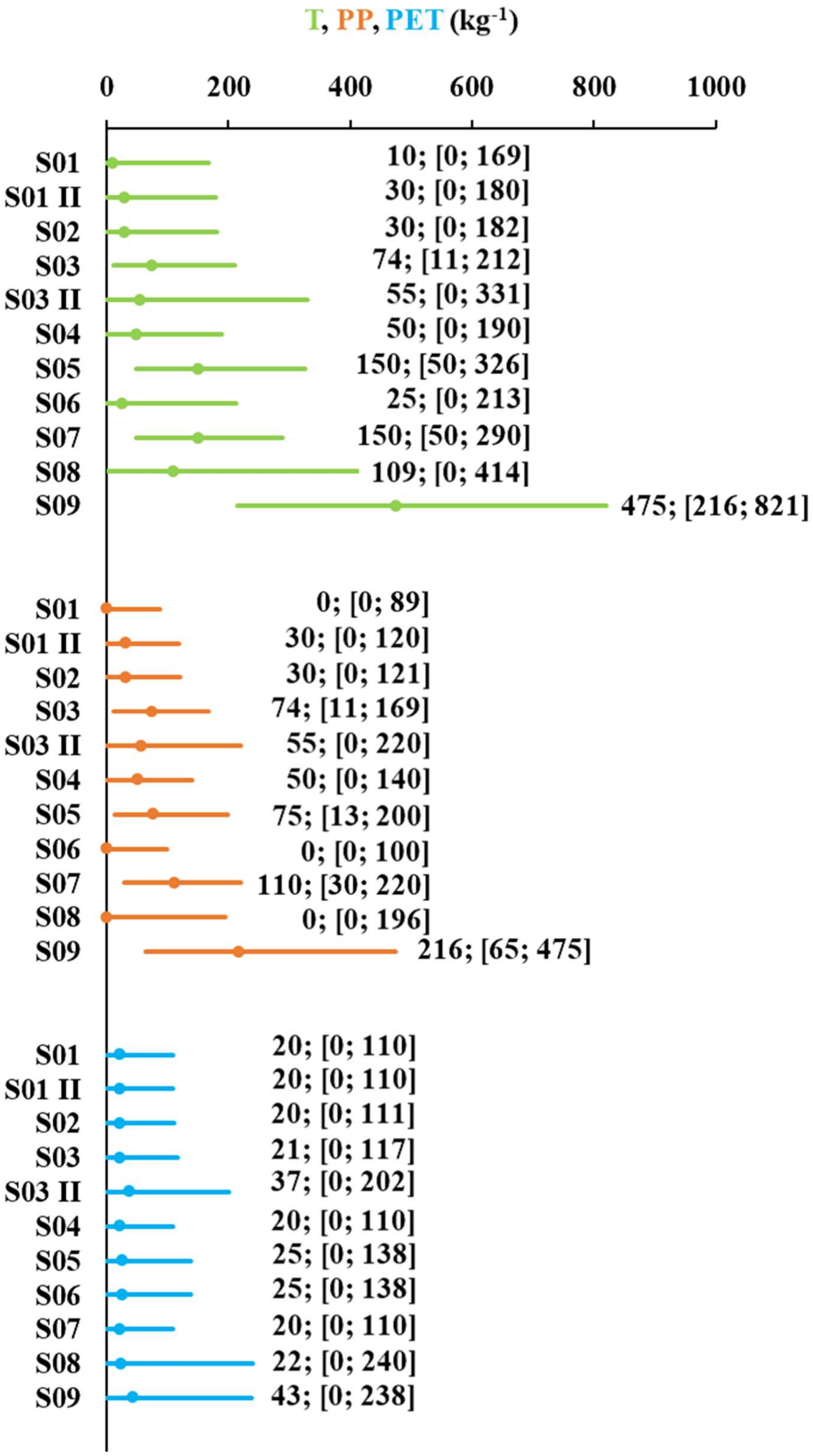

Figure S4. Mode and confidence interval, for 99% confidence level, of simulated estimates of microplastic contamination (T – microplastics less dense than saturated NaCl solution, PP – micro-fragments of polypropylene and PET – polyethylene terephthalate with different shapes) of the samples collected in the first campaign of Mondego River (RMo1). Confidence limits are defined by the 0.5th and 99.5th percentiles (*P*0.5 and *P*99.5, respectively).

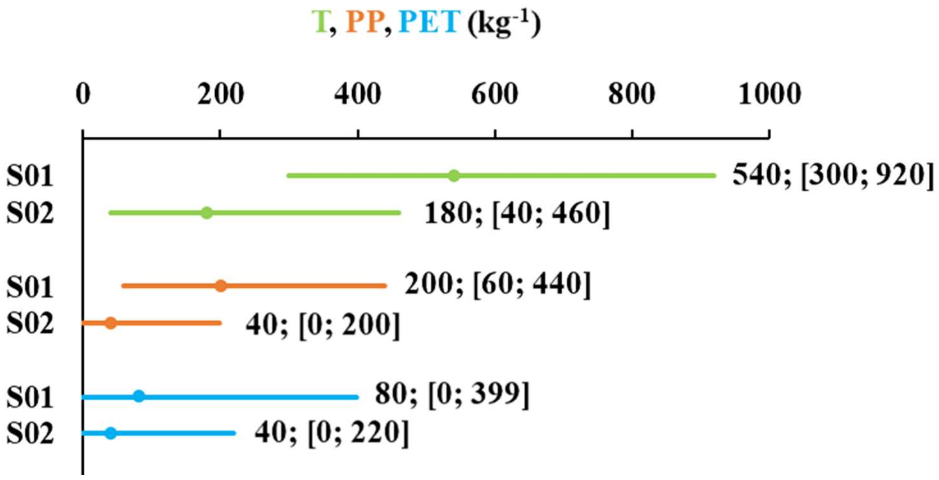

Figure S5. Mode and confidence interval, for 99% confidence level, of simulated estimates of microplastic contamination (T – microplastics less dense than saturated NaCl solution, PP – micro-fragments of polypropylene and PET – polyethylene terephthalate with different shapes) of the samples collected in the second campaign of Mondego River (RMo2). Confidence limits are defined by the 0.5th and 99.5th percentiles (*P*0.5 and *P*99.5, respectively).

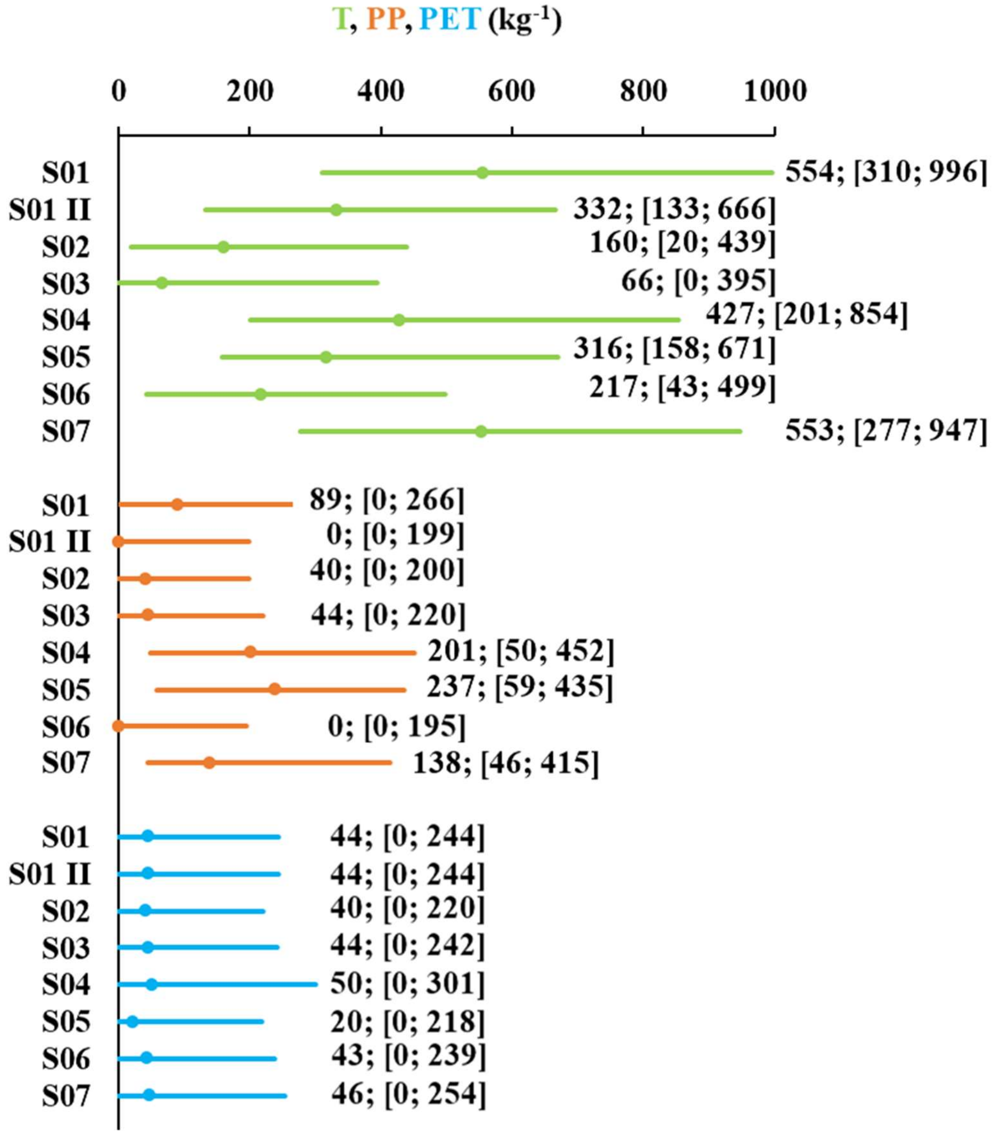

Figure S6. Mode and confidence interval, for 99% confidence level, of simulated estimates of microplastic contamination (T – microplastics less dense than saturated NaCl solution, PP – micro-fragments of polypropylene and PET – polyethylene terephthalate with different shapes) of the samples collected in the first campaign of Mira River (RMi1). Confidence limits are defined by the 0.5th and 99.5th percentiles (*P*0.5 and *P*99.5, respectively).

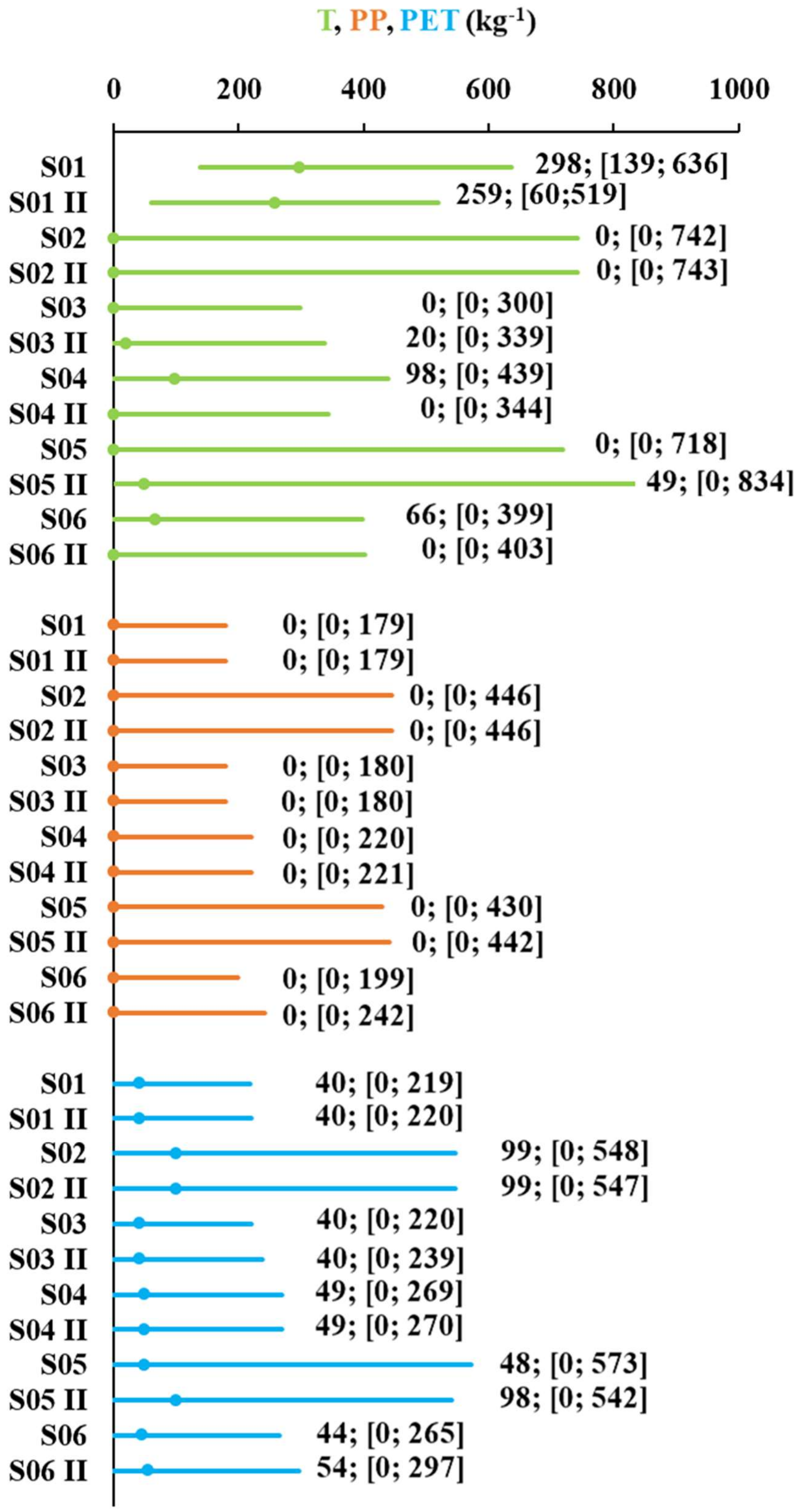

Figure S7. Mode and confidence interval, for 99% confidence level, of simulated estimates of microplastic contamination (T – microplastics less dense than saturated NaCl solution, PP – micro-fragments of polypropylene and PET – polyethylene terephthalate with different shapes) of the samples collected in the second campaign of Mira River (RMi2). Confidence limits are defined by the 0.5th and 99.5th percentiles (*P*0.5 and *P*99.5, respectively).
